# Characterization of Soft Tooling Photopolymers and Processes for Micromixing Devices with Variable Cross-Section

**DOI:** 10.3390/mi11110970

**Published:** 2020-10-29

**Authors:** J. Israel Martínez-López, Héctor Andrés Betancourt Cervantes, Luis Donaldo Cuevas Iturbe, Elisa Vázquez, Edisson A. Naula, Alejandro Martínez López, Héctor R. Siller, Christian Mendoza-Buenrostro, Ciro A. Rodríguez

**Affiliations:** 1 Tecnologico de Monterrey, Escuela de Ingeniería y Ciencias, Monterrey 64849, Mexico; habc-8@hotmail.com (H.A.B.C.); A00831116@itesm.mx (L.D.C.I.); elisa.vazquez@tec.mx (E.V.); A00825462@itesm.mx (E.A.N.); christian.mendoza@tec.mx (C.M.-B.); 2 Laboratorio Nacional de Manufactura Aditiva y Digital (MADiT), Apodaca, Nuevo Leon 66629, Mexico; 3 Centro de Investigación Numericalc, 5 de mayo Oriente 912, Monterrey 64000, Mexico; alex@numericalc.org; 4 Department of Mechanical Engineering, University of North Texas, 3940 N. Elm. St., Denton, TX 76207, USA; Hector.Siller@unt.edu

**Keywords:** micromixers, split-and-recombine, additive manufacturing, surface metrology, asymmetric split-and-recombine (ASAR), stereolithography, surface roughness, soft tooling

## Abstract

In this paper, we characterized an assortment of photopolymers and stereolithography processes to produce 3D-printed molds and polydimethylsiloxane (PDMS) castings of micromixing devices. Once materials and processes were screened, the validation of the soft tooling approach in microfluidic devices was carried out through a case study. An asymmetric split-and-recombine device with different cross-sections was manufactured and tested under different regime conditions (10 < *Re* < 70). Mixing performances between 3% and 96% were obtained depending on the flow regime and the pitch-to-depth ratio. The study shows that 3D-printed soft tooling can provide other benefits such as multiple cross-sections and other potential layouts on a single mold.

## 1. Introduction

Microfluidic-based devices tend to operate under laminar flow regimes where reagent mixing is a significant challenge [1]. Bringing together two separate fluid streams from opposite directions [2] is a strategy that researchers have employed to enhance mixing in a group of devices identified as Split and Recombine (SAR). While the simplest type of SAR device, constituted by a system where two streams collide downstream (T-mixer), has limited performance, further configurations of this principle have been implemented successfully with more intricate geometries, such as rhomboids [3,4], right angles [5,6,7], and arcs [8,9,10,11], as well as the introduction of pillars [12]. Most SAR micromixer devices have been fabricated using approaches such as (a) soft lithography plus polydimethylsiloxane (PDMS) casting, (b) micromachining, or (c) laser ablation.

The development of micromixing has made great strides toward improved designs with better performance and functionality. Hence, SAR micromixers have evolved to adopt more complex three-dimensional structures that include ridges and modular designs [13,14,15,16]. For example, Chen et al. used an array of triangular baffles with three depths to produce transverse movement of fluids toward a cascaded splitting and combination (C-SAR) [17], while Gidde et al. introduced a new design based on rectangular baffles, triple split and recombination (RB-TSAR), and elliptical-based triple split and recombination (EB-TSAR) [18]. Raza and Kim [19] proposed an improved asymmetric split and recombine design based on semi-circular profiles [20] by adding forward and backward-facing steps along the microchannel. They report mixing capabilities of 86% under Reynolds number (Re) below 20.

Recent developments include micromixers based on stacking and folding channels, showing a mixing efficiency beyond 95% [21], as well as good performance under a wide range of Reynolds number (0.5 < *Re* < 100) for Newtonian and non-Newtonian fluids [22]. These complex designs are fabricated via micromilling [21,22,23]. Other advanced micromixers are based on serpentines with non-rectangular cross-sections [24,25].

The toolkit of materials and manufacturing technologies available for microdevice designers has expanded significantly using additive manufacturing [26,27,28]. For example, Shallan et al. [29] manufactured a three-dimensional micromixer design through direct 3D printing. The concept is based on a 10× scaled-up version (500 µm) of a three-dimensional mixer based on the Baker Map on a theoretical work developed by Carriere [30]. The design is a paragon of the ideal micromixer device. It represents a three-dimensional projection of the split and recombine concept within all the Cartesian directions and mixing at perpendicular angles. Nonetheless, direct device manufacturing (one-step) is still challenging due to the difficulty of 3D printing internal channels [31].

The rapid or soft tooling approach, initially developed for a low-volume production environment, is a viable alternative when complex microdevices are required [32]. Depending on the application, additive manufacturing can be applied directly or indirectly to build tooling (molds). With a direct tooling approach, a mold or die is created directly through 3D printing [33,34]. Complex micromixers with convoluted features can provide an improved homogeneity of samples and tackle the limitations of the low Reynolds number regime [35].

Stereolithography (SLA) or vat photopolymerization comprises several additive manufacturing processes that rely on the selective curing of resin using a UV light. SLA can provide the UV light through several methods, including a laser, a digital micromirror device (DMD), or a combination of a lamp and alLiquid Crystal Display (LCD) based mask. Compared to most 3D printing processes, SLA provides fine resolution, in a range sufficient to reproduce the designs of intricate three-dimensional micromixers. While SLA brings a wide range of possibilities in the development of novel microfluidic devices [36], the available literature is limited in terms of testing and validation of process capability and reliability.

In the past, our group has shown the potential of soft tooling [37]. The objective of this study is to characterize a wider range of photopolymers and associated stereolithography processes in the context of good manufacturing practices for the development of micromixing devices. Surface and dimensional metrology were carried out for soft tooling (molds) in order to assess the process capability and potential chemical interaction with the cast PDMS. Once the photopolymers and SLA processes were screened, an asymmetric split-and-recombine (ASAR) device was built as a case study.

## 2. Materials and Methods

### 2.1. Soft Tooling Material Process Screening

**Figure 1:**
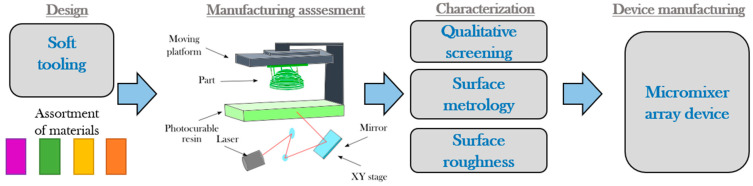
Steps for the screening of soft tooling based on stereolithography.

Figure 1 describes the process to develop micromixing devices with complex features followed in this work. The design of molds of microdevices that feature a varying cross-section is introduced toward developing complex devices that are more prone to be adapted in-field. The new design introduces the capability to cast devices with multiple cross-sections on a single step toward developing complex devices that are more prone to be adapted in-field. The steps aim to examine an assortment of materials in three different types of stereolithography-based additive manufacturing processes. The materials were selected considering that the employment of resins had become an affordable benchtop technology available for microdevice designers in their lab or as a service [38,39,40,41]. Each material’s screening is performed with surface characterization and dimensional metrology of selected features and evaluating the usability and the potential to produce functional devices. Insights of the screening can then be applied for the soft tooling of improved devices.

#### 2.1.1. Screening Mold Geometry

**Figure 2:**
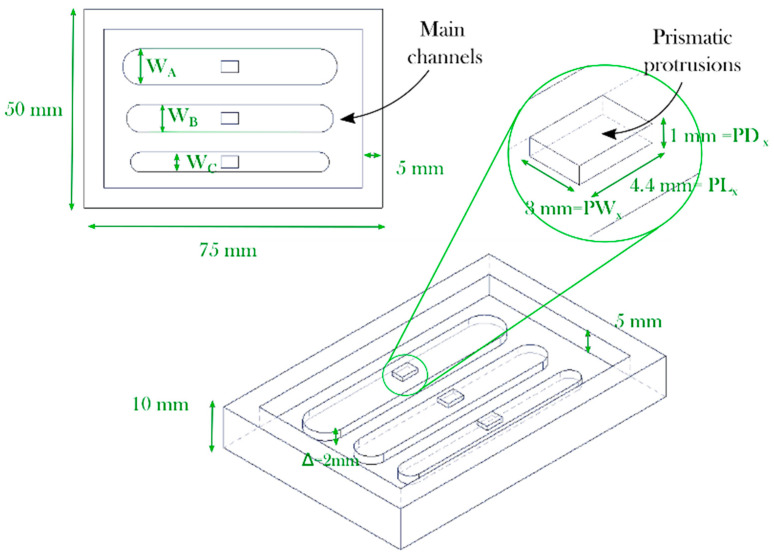
Soft tooling design for photopolymers and process screening.

A mold with three main channels and protrusions was designed for material screening (see Figure 2). A depth of 5 mm was selected for the main chamber. Each mold is patterned with three main channels (*W*_A_ = 9 mm, *W*_B_ = 7 mm, and *W*_C_ = 5 mm) and a singular depth or pitch (∆ = 2 mm).

#### 2.1.2. Qualitative Assessment

The assortment of materials and additive manufacturing techniques were tested experimentally as soft tooling alternatives with the conditions shown below. The test consisted of employing simple manual tools including tweezers, an X-Acto knife, and a Silhouette toolkit that included a hook and a scraper (Silhouette America, West Orem, UT, USA). Once each mold was developed, observations were made to record the usability of the device compared with an aluminum device. The examination of each process included evaluating the difficulty of removing the PDMS elastomer from the device and reviewing the existence of any abnormality observed in the molded pattern after the curation process.

#### 2.1.3. Screening Mold Metrology

For the characterization of the molds, an Alicona Infinite Focus Measurement Machine (Bruker Alicona Headquarters, Graz, Austria) was selected. This device allows for the acquisition of datasets at a high depth of focus, similar to a Scanning Electronic MicroscopeSurface roughness (*Ra*), measured using a 10x optical lens.

#### 2.1.4. Aluminum Mold Manufactured Using a Conventional Subtractive Methodology

A mold was manufactured with conventional subtractive methodology using an aluminum alloy and a 3-axis vertical milling machine center Makino F3 (Makino Inc., MASON, OH, USA) with a maximum spindle speed of 30,000 RPM. A tool holder and a clamp were used for holding the workpiece. A laser measuring system by BLUM (Blum-Novotest Ltd., Staffordshire, UK) was used for tool setting. Three solid tungsten carbide cutting tools were used; a 12.7 mm diameter flat end mill with 4 flutes, a 5 mm diameter flat end mill with two flutes, and a 3 mm flat end- mill with 2 flutes were used for rough, semi-finish, and finish operation. Given the close tolerances achievable through machining, this mold was used as a reference base line.

### 2.2. Additive Manufacturing Processes and Materials

A professional laboratory equipment DLP-SLA Envisiontec Perfactory P3 Mini Multilens, that uses a 60 mm lens system, a work tray of 84 × 63 × 230 mm, and a layer resolution between 15 µm and 150 µm was employed to generate the molds using ABS Flex White, HTM 140, E-Dent 400, ABS Flex Black, and E-Partial. These materials were post-processed in an Otoflash pulse curing chamber of the same supplier (11 W lamp with a wavelength between 300 and 700 nanometers and ten pulses per second).

A benchtop SLA-LF Form 3 additive manufacturing equipment from Formlabs was used employing a 25 µm for the Clear V04 and 50 µm resolution setting for the Flexible V02 resin. Samples were cured using the provider’s recommended settings for post-processing the sample accordingly (15 min at 60 °C and 1 h of exposure to UV light).

Additionally, a 3D Systems ProJet 6000 that works with an ultraviolet laser was employed by a service provider [42] to develop devices in Accura SL 5530 resin. The thermoset resin selected for the molds was a high-temperature resistant stereolithography material, Accura SL 5530 (3D Systems, Rock Hill, SC, USA). A post-curing process was performed to the mold by exposing it for 90 min to UV light and baking it at a temperature of 160 °C for 2 h.

### 2.3. Microdevice Polydimethylsiloxane (PDMS) Casting

The standard process for PDMS casting was followed in this work. First, the Sylgard 184 PDMS (Dow-Corning, Midland, Michigan) was poured into a vessel and mixed with a curing agent in a 10:1 ratio by weight. To control this ratio the material was weighed using a calibrated analytical balance (Mettler AT200, Columbus, OH, USA). Then, the mixture was exposed to a vacuum chamber for 10 min to eliminate most of the air bubbles produced before and after pouring the material into the mold. Finally, each mold was placed over a hot plate at 75 °C for polymerization. The temperature was set below the glass transition temperature overnight (12 h) for the process screening and 45 min for the case study. In the latter, shorter times were considered to recreate a more lifelike and time demanding application. For other materials or experimental setup, different times might be required. The PDMS parts were removed using manual tools such as a cutting knife and tweezers.

For the case study, the inlets and outlet ports were punched using a 1.25 mm (internal diameter) Miltex disposable biopsy plunger (Integra Life Sciences, Princeton, NJ, USA). Then, the part was bonded to a 76 × 52 × 2 mm glass slide (VWR International, Radnor, PA, USA). Once the glass slide was cleaned with water and ethanol, the parts were introduced into a plasma cleaner (Harrick Plasma Inc., Ithaca, NY, USA). A vacuum inside the chamber was created for 3 min and the parts were exposed to plasma for 3 min. After three (3) more minutes, the treated surfaces were put in contact for PDMS-glass bonding. A visual inspection of the device was carried out to verify that the device was properly bonding and without bubbles trapped inside.

### 2.4. Case study: ASAR Micromixer Array

#### 2.4.1. Micromixing Mold Geometry

**Figure 3:**
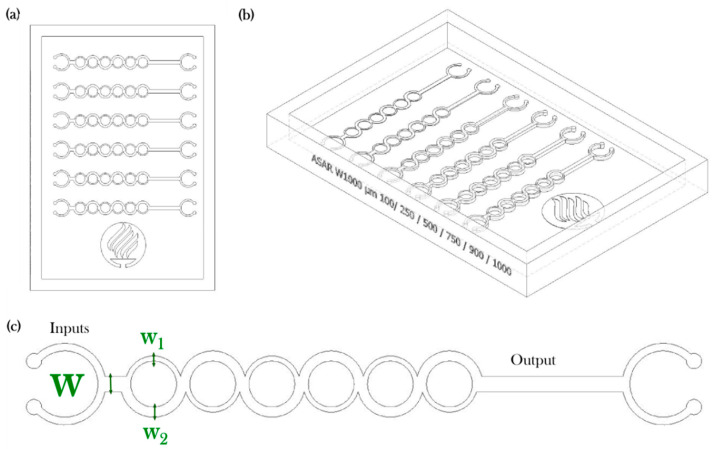
Soft tooling case study: (**a**) Top view, (**b**) Isometric view, (**c**) Detail of micromixer.

The mixing performance of the proposed microdevice is based on the one developed by Ansari et al. [17] and used by our research group in the past [43,44]. This version has the added complexity of a variable depth or pitch **∆** (see Table 1 and Figure 3a). The device is composed of a single channel of 1000µm that splits into two subchannels of uneven width and converges on a channel six times (see Figure 3b,c). A set of micromixer molds were manufactured using the parameters described in Section 2.1 for ABS Flex White Resin. Additional molds were manufactured using ABS Flex Black and Accura SL 5530 resins.

#### 2.4.2. Micromixing Performance Evaluation

Reynolds number is conventionally used to characterize the behavior of the flow conditions within microdevices and is defined as the ratio of inertial to viscous forces. Equation (1) represents the Reynolds number (*Re*) defined as:

(1) $$Re=\frac{inertial\;forces}{viscous\;forces}=\frac{\rho UD_{h}}{\mu }$$

where *µ* is the viscosity (Pa s), *ρ* is the fluid density (kg m^−3^), *U* is the average velocity of the flow (m s^−1^), and *D_h_* is the hydraulic diameter of channel (m), which is defined in Equation (2) as:

(2) $$D_{h}=\frac{4A}{P}=\frac{4w*\Delta }{2w+2\Delta }$$

where *A* and *P* are the area and the wetted perimeter of the cross-section, which is given by the micromixer width (*W*) and depth (∆).

To quantify the mixing behavior, the variance of the liquid species in the micromixer (*σ*) was calculated. The variance of the species was determined at the cross-sectional area at the output of the micromixer perpendicular to the x-axis. To evaluate the degree of mixing, the variance of the mass fraction of the mixture in a cross-section (*σ*) that is normal to the flow defined in Equation (3):

(3) $$\sigma =\sqrt{\frac{1}{N}{\left(c_{i}-{\bar{c}}_{m}\right)}^{2}}$$

where *N* is the number of sampling points inside the cross-section, *c_i_* is the mass fraction at the sampling point I, and *c_m_* is the optimal mixing mass fraction, which is 0.5 at any cross-sectional plane (ideal mixing). To quantitatively analyze the numerical mixing performance of the micromixer, the mixing index (*M*) at a cross-sectional plane is shown in Equation (4), which can be defined as:

(4) $$M=1-\sqrt{\frac{\sigma^{2}}{\sigma_{max}^{2}}}$$

where the mixing efficiency (ranges from 0.00 (0% mixing) to 1.00 (100% mixing). The maximum variance *σ_max_* represents a completely unmixed condition.

#### 2.4.3. Micromixing Experimental Setup

An experimental setup for testing mixing efficiency was used for micromixing evaluation. Inlet flows were programmed in a syringe pump (KDS200, Holliston, MA, USA). The micromixer device was evaluated on a range of conditions using the blue dye and distillate water under an Axiovert 200 inverted microscope (Carl Zeiss AG, Oberkochen, Germany). The mixing measures were calculated using intensity profiles. The image processing was performed using the custom software MIQUOD (Mixing Quantification of Devices). The actual flow entering the channels varied considering the hydraulic radius of each of the microchannels, and the Reynolds number at which the micromixer was tested was calculated using the FS3 and FS5 flow meters (Elvesys, Paris, France) connected to the MFS flow reader (Elvesys, Paris, France). The acquisition of 1920 × 1080 pixels images was made using a C930E webcam (Logitech Incorporated, Newark, CA, USA).

The ASAR micromixer array with a variable depth was developed using an Accura SL 5530 resin and tested to assess how relevant the channel pitch for determining the mixing performance. The target areas were equally defined for all the measures (193 × 197 pixels) and calibrated with a channel completely filled with dye and with water before experimentation. The calibration was carried out under the same operating conditions in the experiment (micromixer position and lighting).

## 3. Results

### 3.1. Qualitative Assessment of Screened Materials

The qualitative observations derived from the testing of materials for 3D printed mold are shown in Table 2. All the molds were tested under similar conditions. From the assessment it was observed that only the aluminum, E-Partial, ABS Flex white, Clear V04, and Flexible V02 showed the potential for the development of microfluidic devices. In contrast, E-Dent 400, ABS Flex Black, and HTM140 are not recommended for the development of molds. Difficulties for the remotion and swelling have been reported before for untreated bulk and surface of PDMS due to incompatibility with solvents, the absorption of small molecules, or deformations during casting [45,46,47]. It is possible that these could be some fundamental reasons for the negative observations registered.

PDMS is a kind of silicone. The curing of silicones can be inhibited when in contact with materials that contains sulfur, tin, and nitrogen [48]. In the case of HTM 140, E-Dent 400, and ABS Flex Black, a partial or significant PDMS curing inhibition was found. Therefore, these materials are not recommended as molds. While ABS Flex White resin and ABS Flex Black share the same mechanical properties, it is suspected that the pigment on the latter inhibited PDMS curing. Unfortunately, vendors of 3D printing materials as the ones used in this study do not provide details on the chemical composition and additives. Therefore, it is difficult to provide a detailed analysis as to the reasons behind curing inhibition problems. In addition to the adverse effect of PDMS curing inhibition, the printed mold flexibility plays a significant role in the demanding process of the cast PDMS, as indicated in Table 2, that ranks mold materials from best to worst.

Once this initial qualitative screening was conducted, Clear V04 and Accura SL 5530 were used to produce cast PDMS components. With both of these materials, the plasma treatment was successful when sealing the PDMS component to the microscope slide.

**Figure 4:**
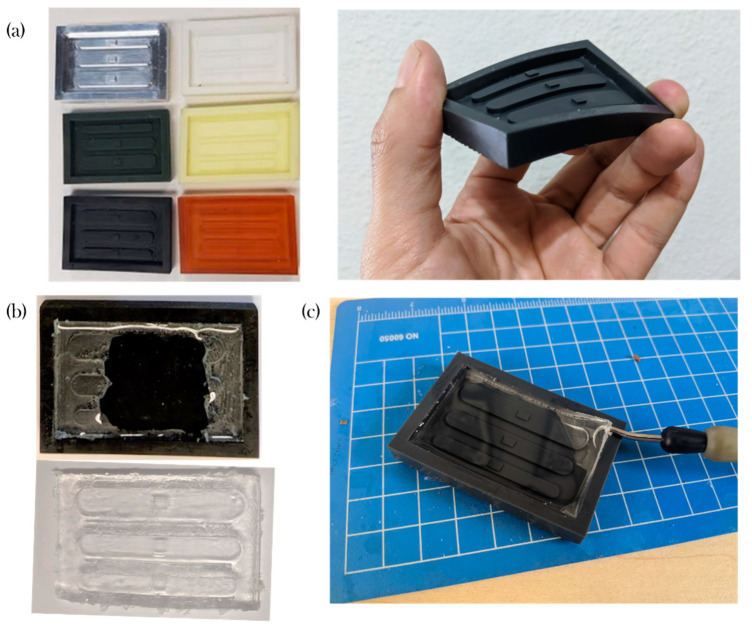
Assessment of material compatibility; (**a**) Picture of sample molds); (**b**) Demolding difficulties; (**c**) Polydimethylsiloxane (PDMS) slab removal on a Flexible v02 mold.

Figure 4a shows a picture of some of the molds manufactured. The materials that are listed with difficulties for demolding either presented bad surface quality or the part could not be removed (See Figure 4b). This problem was most frequently observed between channels B and C and between channel C and the edge of the device, considering that these were the narrowest features.

Compared to the aluminum mold, the polymeric devices were prone to breaking due the force induced by the manual tools. For example, while the Accura SL 5530 resin mold produced a patterned part, one of the walls was shattered during the extraction.

In contrast, compelling results on the usability of the flexible material (Flexible v02) resin were found as the patterned piece could be pulled easily without apparent risks of destroying the device.

### 3.2. Dimensional and Surface Metrology of Photopolymers and AM Processes

Infinite Focus Microscopy (IFM) has been shown to be capable of capturing images with a lateral resolution down to 400 nm providing 3D data sets with very accurate results [49]. Table 3 and Table 4 resume the dimensional and surface metrology of the prismatic protrusions and main channels.

While the deviational error of the aluminum mold using subtractive manufacturing is still a paragon, there are some major advantages on the surface quality of the metallic device compared with the polymeric counterparts, however, such as low surface roughness and a significantly higher bulk modulus (easing the removal of the part from the mold). Low-force stereolithography printing uses a flexible tank and linear illumination to deliver and improve surface quality and print accuracy. According to the provider, lower print forces allow for support structures to be removed more easily (compared to traditional stereolithography) [50].

**Figure 5:**
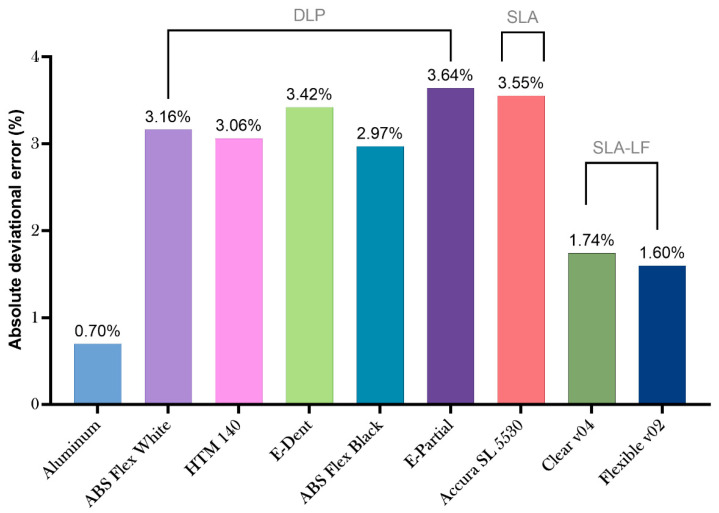
Absolute deviational error of assessed materials.

Figure 5 Clear V04 and Flexible V02 offered the closest (and lowest) deviational error to the aluminum molds among all the materials. The other materials had an absolute deviational error around between 2.97% and 3.64%. Additional data is available in the Supplementary Material.

**Figure 6:**
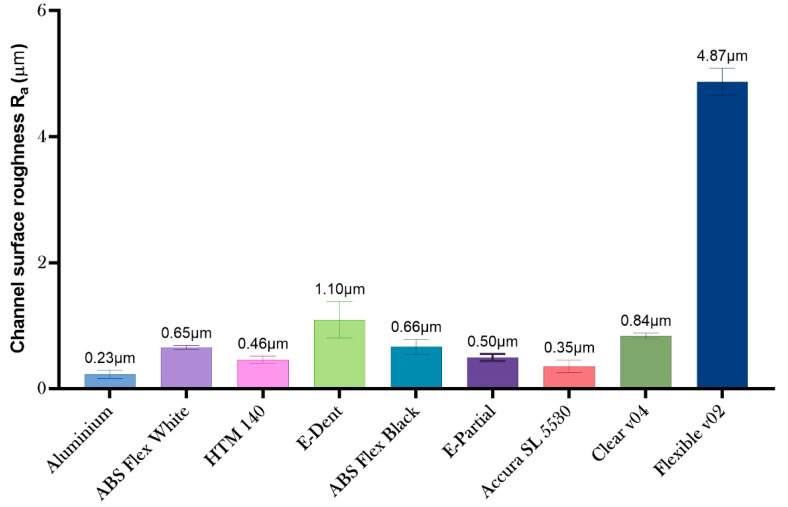
Mean surface roughness of assessed materials.

In terms of the surface roughness of the devices, the values were very similar among the prismatic protrusions and the channels (see Table 3 and Table 4). The average surface roughness of each material is shown in Figure 6. The roughest surface was the Flexible v02, and the smoothest molds were produced with the Accura SL 5530 and the HTM 140 resins.

### 3.3. Case Study: Asymmetric Split-and-Recombine (ASAR) Microdevice Surface Metrology

An additional evaluation of features was done for some of the screened materials to evaluate if the features of a device with finer features could be produced and screened with a similar methodology as the molds made in Section 2.1.1.

**Figure 7:**
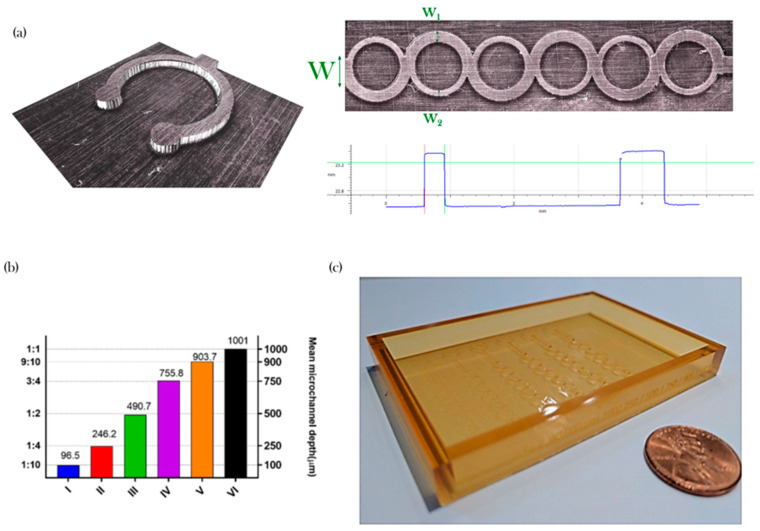
Micromixing device metrology; (**a**) Measurement of a key feature; (**b**) Mean microchannel pitch vs to width ratio and (**c**) Picture of an asymmetric split-and-recombine (ASAR) soft tool mold.

Figure 7 shows examples that highlight the advantages of Infinite Focus for evaluating the features compared with other metrology options, such as a stylus-based profilometer. With this methodology, it is possible to obtain details in the micro and meso-scale concurrently in three-dimensional models and determine important functional features such as the width of the subchannels of the ASAR micromixer (Figure 7a).

Overall, the manufacture using the materials was consistent with the data described above. For example, the characterization of the set of ABS Flex White showed to be below 10 µm (see Figure 7b). Figure 7c shows an example of a device manufactured using the proposed methodology and employed for the micromixing performance manufactured with Accura SL 5530 (see Section 3.4). The material was selected because it displayed more potential for usability (see Table 2).

### 3.4. Micromixing Performance

**Figure 8:**
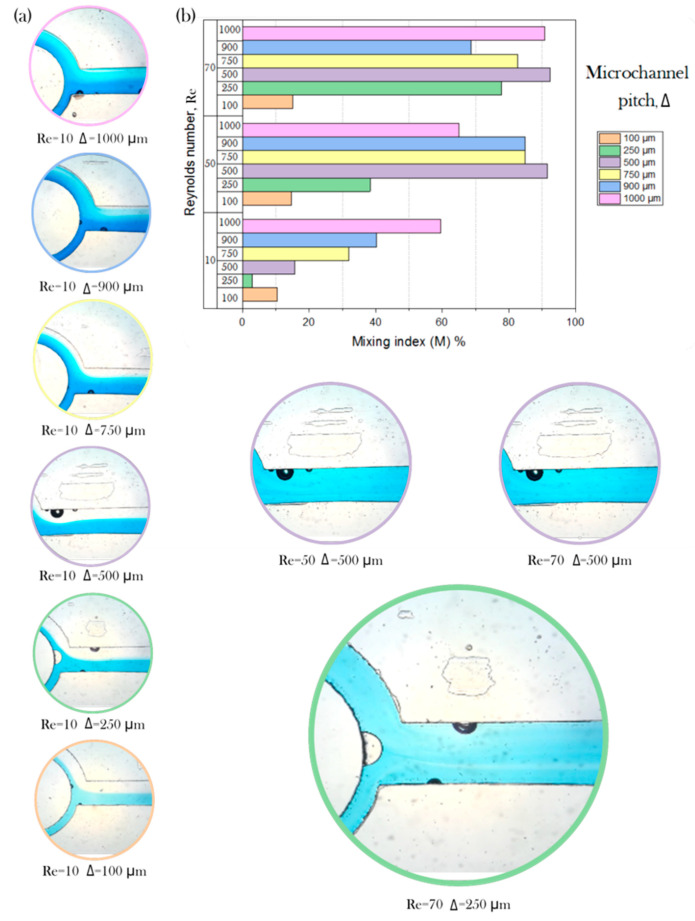
Case study; (**a**) Channel depth assessment comparison using the calculated mixing indexes in a range of 10 < Re < 70; and (**b**) Mixing index M vs Reynolds number vs microchannel pitch (Δ).

Pictures obtained with the experimental setup described in Section 2.4.3 are shown in Figure 8a as examples of the varying conditions on the degree of mixing, depending on the Reynolds number and the pitch Δ. Figure 8b describes the mixing index (*M*) values quantitatively. As expected, a higher Reynolds number can be associated with a higher degree of mixing overall, considering that in conditions where the inertia forces are more predominant then the viscous forces, the chaotic advection can act to create secondary vortices and enhance mixing downstream.

The disposition of the array allowed us to evaluate the performance of the device under similar conditions for different depths and hence different width-to-height ratios (see Figure 8b). Notice that the color of the circle around the picture indicates the corresponding pitch (orange for 100 µm, green for 250 µm, purple for 500 µm, yellow for 750 µm, blue for 900 µm, and pink for 1000 µm). An examination of the data suggests that increasing the microchannel pitch can help increase the degree of mixing. However, the mixing performance improvement between the flow regime of Reynolds number 50 and Reynolds number 70 is limited. During the process, some bubbles were formed on some of the channels (*Re* = 70 and Δ = 250 µm); however, these appeared adjacently to the microdevice walls, and a disruption of the flow mixing due was not observed. The mixing performance showed a dependence on the pitch of the microchannel and the Reynolds number.

The capability to modify the mixing efficiency using a ratio of a single feature to the pitch of the channel has been reported previously [51,52]. Graph (Figure 8b) shows an upward trend between pitch and mixing efficiency. While the ASAR device was developed successfully, there are limitations that offer opportunities for further research to comprehend the phenomena underlying the operation.

### 3.5. Learned Lessons and Future Work

The applications of the learned lessons can be resumed in the case study as follows:

- It is possible to produce different versions of the same device on a single mold with a single demolding step. Other possible layouts include different devices on a single molding step or an array of a single device.
- Device identifier: engraving symbols on the device can be implemented for identifying the mold among different variations.
- Other potential futures were prospected for future work, as removable wall(s) that could ease the remotion of the PDMS and the capability to dispose placeholders for inlet or outlet pins as part of the mold.

## 4. Conclusions

The concluding remarks of this work are summarized as follows:

- The rapid or soft tooling approach was screened for eight (8) different photopolymers as a viable option for developing complex micromixing devices.
- The experimental data provided valuable insights on acceptable manufacturing practices toward a new generation of devices.
- The novel design of the mold with variable depth was successfully implemented to test different regime conditions within the same device.
- Methodology for the production of an array of micromixers with a variable cross-section was successfully implemented.
- Multiple cross-sections on a single device could be implemented using stereolithography. Other device setups (an array of a single device or different type of devices on a single mold) could be implemented using the methodology presented in this work.
- Surface characterization showed an absolute deviational error within 10 micrometers.
- Stereolithography is a viable option for the development of complex three-dimensional molds for the development of micromixers, but it is necessary to consider the surface-to-surface interaction between the mold and the resin.
- Further studies are required to evaluate the effect of the geometrical features of the ASAR micromixer thoroughly.

## Figures and Tables

**Table 1:** Micromixers array employed to assess the manufacturing technology.

Device Type	Array Elements	Microchannel Width (*W*)	SAR Subchannels (*W*_1_/*W*_2_)	Micromixer Pitch ∆
Asymmetric split and recombine micromixer	6	1000 µm	667/333 µm	I = 100 µm, II = 250 µm, III = 500 µm, IV = 750 µm, V = 900 µm, VI = 1000 µm

**Table 2:** Qualitative assessment of photopolymers and additive manufacturing processes.

Material	Manufacturing	Tensile Strength/Modulus (MPa) ^1^	Observations during Casting
Aluminum 7075	Micromilling	276/ 68900	Excellent surface quality, easy demolding
Clear V04	SLA-LF	65/2800	Excellent surface quality, excellent demolding
Accura SL 5530	SLA	57-63/2854-3130	Good surface quality
E-Partial	SLA-DLP	129/3125	Good surface quality
ABS Flex White	SLA-DLP	65/1772	Good surface quality
Flexible V02	SLA-LF	3.4/8.5	Fair surface quality, easy demolding due flexibility
* E-Dent 400	SLA-DLP	85/2100	Difficulties for demolding
* ABS Flex Black	SLA-DLP	65/1772	Difficulties for demolding
* HTM 140	SLA-DLP	115/3350	PDMS reaction

^1^ Information from datasheets. * Note: these resins are not recommended.

**Table 3:** Dimensional and surface metrology of prismatic protrusions.

Feature	Feature	Al	ABS Flex White	HTM 140	E-Dent 400	ABS Flex Black	Accura SL 5530	Clear V04	Flexible V02
PD (mm)	Protrusion depth	1.006 ± 0.002	1.014 ± 0.013	1.026 ± 0.003	0.943 ± 0.002	1.011 ± 0.009	0.890 ± 0.013	1.006 ± 0.011	0.993 ± 0.011
PL (mm)	Protrusion length	4.437 ± 0.002	4.207 ± 0.040	4.217 ± 0.052	4.512 ± 0.024	4.202 ± 0.019	4.450 ± 0.02	4.449 ± 0.041	4.504 ± 0.100
PW (mm)	Protrusion width	3.036 ± 0.002	2.890 ± 0.013	2.887 ± 0.036	3.032 ± 0.012	2.908 ± 0.006	3.11 ± 0.056	3.02 ± 0.009	3.083 ± 0.010
*Ra* (µm)	Protrusion roughness	0.262 ± 0.085	0.636 ± 0.002	0.526 ± 0.105	0.91 ± 0.170	0.394 ± 0.042	0.303 ± 0.036	0.807 ± 0.098	4.833 ± 0.164

**Table 4:** Dimensional and surface metrology of main channels.

Feature	Feature	Al	ABS Flex White	HTM 140	E-Dent 400	ABS Flex Black	Accura SL 5530	Clear V04	Flexible V02
W_A_ (mm)	Channel width A	5.038	4.484	4.758	5.001	4.819	5.035	9.056	9.098
W_B_ (mm)	Channel width B	7.044	6.634	6.642	6.884	6.714	7.072	7.029	7.15
*W*_C_	Channel width C	9.035	8.574	8.552	8.796	8.642	9.104	5.07	5.056
D_ABC_ (mm)	Channel depth	1.996 ± 0.004	1.966 ± 0.015	2.004 ± 0.011	1.871 ± 0.008	1.954 ± 0.004	2.015 ± 0.005	2.009 ± 0.013	1.970 ± 0.010
*Ra* (µm)	Channel Roughness	0.225 ± 0.066	0.653 ± 0.032	0.460 ± 0.056	1.095 ± 0.288	0.664 ± 0.120	0.354 ± 0.10	0.839 ± 0.043	4.871 ± 0.214

## Supplementary Materials

The following are available online at https://www.mdpi.com/2072-666X/11/11/970/s1, Supplementary file S1: Dimensional and surface metrology of materials for stereolithography-based AM processes.
